# Laser treatment for Cafe-au-lait Macules: a systematic review and meta-analysis

**DOI:** 10.1186/s40001-023-01143-1

**Published:** 2023-06-08

**Authors:** Zi-Zhen Guo, Zhi-Chao Wang, Dun Wang, Ling-Ling Ge, Yue-Hua Li, Yi-Hui Gu, Wei Wang, Cheng-Jiang Wei, Bin Gu, Min Yao, Ji-Ying Dong, Qing-Feng Li

**Affiliations:** 1 Department of Plastic and Reconstructive Surgery, Shanghai Ninth People’s Hospital, Shanghai Jiao Tong University School of Medicine, 639 Zhizaoju Road, Shanghai, 200011 People’s Republic of China; 2 Department of Burn and Plastic Surgery, West China Hospital of Sichuan University, No 37 Wainan Guoxue Road, Chengdu, 610041 China

**Keywords:** CALMs, Laser treatment, Systematic review and meta-analysis

## Abstract

Nowadays, laser is the mainstay treatment for cafe-au-lait macules (CALMs), but no systematic review has been published to demonstrate the overall efficacy and it’s still controversial which type of laser is optimal. Thus, we conduct the meta-analysis to evaluate the effectiveness and side effects of various types of lasers in treating CALMs. Original articles reporting the efficacy and side effects for CALMs in laser treatment were identified in PubMed, EMBASE, and Web of Science from 1983 to April 11, 2023. Using R software and the ‘meta’ package, meta-analysis was conducted for clearance and recurrence for evaluation of efficacy. And the occurrence of hypopigmentation and hyperpigmentation rate was pooled for safety evaluation. We used RoB2 and ROBINS-I tools to assess the risks of bias in RCT studies and non-RCT studies, respectively. The Grading of Recommendations, Assessment, Development and Evaluation system was used to assess the quality of the evidence. Nineteen studies involving 991 patients were included, which had a very low to moderate quality of evidence. The pooled 75% clearance rate was 43.3% (95% CI 31.8–54.7%, *I*^2^ = 96%), 50% clearance rate was 75% (95% CI 62.2–85.9%, *I*^2^ = 89%) and the recurrence rate was 13% (95% CI 3.2–26.5%, *I*^2^ = 88%). The pooled hypopigmentation and hyperpigmentation rates were 1.2% (95% CI 0.3–2.1%, *I*^2^ = 0%) and 1.2% (95% CI 0.3–2%, *I*^2^ = 0%), respectively. Subgroup analysis revealed that QS-1064-nm Nd:YAG laser treatment not only achieved more than 75% clearance rate in 50.9% of patients (95% CI 26.9–74.4%, *I*^2^ = 90%) but also resulted in the lowest hypopigmentation and hyperpigmentation rate of 0.5% (95% CI 0.0–2.5%, *I*^2^ = 26%) and 0.4% (95% CI 0.0–2.5%, *I*^2^ = 0%). To draw a conclusion, the laser treatment could reach an overall clearance rate of 50% for 75% of the patients with CALMs, for 43.3% of the patients, the clearance rate could reach 75%. When looking at different wavelength subgroups, QS-1064-nm Nd:YAG laser exhibited the best treatment capability. Laser of all the wavelength subgroups presented acceptable safety regarding of the low occurrence of side effects, namely, hypopigmentation and hyperpigmentation.

## Introduction

Cafe-au-lait macules (CALMs) are pigmented lesions found in 2–3% healthy newborns or patients with genetic diseases, such as neurofibromatosis type 1 [1] and Noonan syndrome [2]. They show as pigmented macules or patches [3]. Histologic changes seen in CALMs are subtle and nonspecific. There is increased basilar melanin pigment with either a normal number of melanocytes or melanocytic hyperplasia [4]. CALMs can be morphologically classified including their size, color, location, and border, which have an effect on their treatment response. Besides, CALMs could be a clue of many different genetic diseases including Neurofibromatosis type 1, Legius syndrome [5] and etc. Although CLAMs don’t have any side effects on health, they may cause a great effect on appearance, leading to low self-esteem and shame, so patients with CALMs always have difficulty to integrate into social life.

Nowadays laser is the mainstay treatment for CALMs. The mechanism of laser or light treatment for pigmented lesions is based on the selective photo-thermolysis theory which is proposed by Anderson et al. in 1983. It proposed that melanin in the epidermal is vaporized and broken by absorbing the energy of the laser so that the laser treatments are able to clean epidermal pigmented lesions [6]. Generally, the double-frequency neodymium-doped: yttrium aluminum garnet (532 nm Nd: YAG) laser, the ruby laser (694 nm), the alexandrite (755 nm) laser, and the neodymium-doped: yttrium aluminum garnet (1064 nm Nd: YAG) laser are most used. In practice, as the wavelength of the laser increases, the light is absorbed less but penetrates deeper. Therefore, the 532 nm and 694 nm are the most appropriate wavelengths for epidermal lesions, followed by the 755 nm and then the 1064 nm wavelength.

However, no clinical consensus has been reached on which wavelength is the best choice for treating CALMs and due to the limited sample size, the strength of the available evidence remains open to question. Thus, we conducted this comprehensive and systematic meta-analysis of published data on the efficiency and side effects of the laser treatment for CALMs. At the end of the article, on the basis of summarizing and thinking about previous studies, we put forward new ideas of CALMs treatment.

## Results

### Study characteristics and risk of bias

**Fig. 1:**
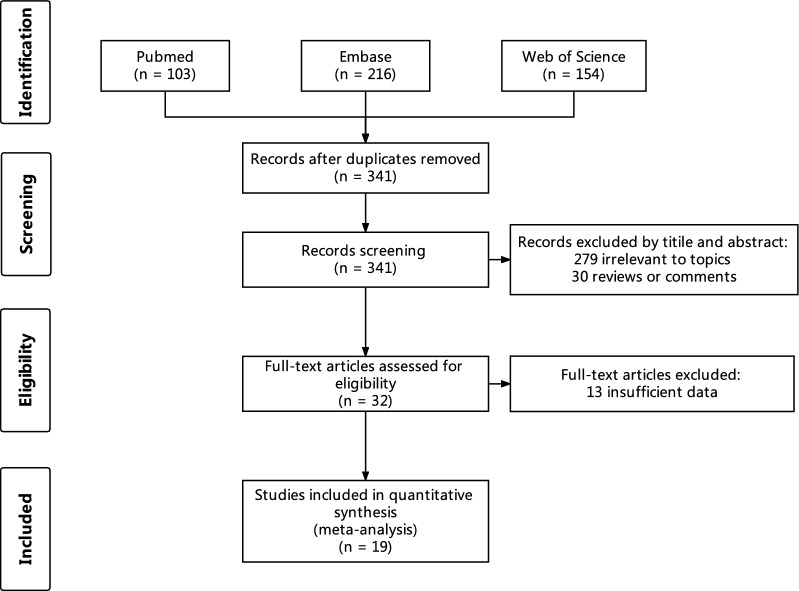
Flowchart showing the process of identification of selected studies

The search strategy initially retrieved 532 potentially relevant clinical studies. A total of 19 studies published between 1983 and April 11, 2023 were included. The flow chart of reference selection is presented in Fig. 1. The general characteristics of studies included in the meta-analysis are presented in Table 1. Two studies were randomized clinical trials (RCT) [7], five studies were retrospective studies [9] and the rest were prospective clinical trials [17]. A total of 991 patients were included in our meta-analysis. The outcomes were evaluated by dermatologists visually and lesion clearance was graded on the visual analog scale (VAS) in 2 studies [7]. As for the side effects, the recurrence rate of CALMs was mentioned in 12 studies and 7 of them were more than 10% [18]. Hypopigmentation and hyperpigmentation were mentioned in 14 studies [14].

**Table 1:** Baseline information of light treatments to CALMs

Authors	Study type	Lesions	Wave-length (nm)	Pulse duration	Sopt size (mm)	Fulence (J/cm^2^)	Session	Interval (week)	Follow-up (month)	Outcomes	Recurrence rate
Kilmer et al. [17]	Prospective	7	QS-532	10 ns	2	2,3,4,5	1	/	3	14% improve > 75%, 57% improve $\le$ 25%	/
Cen et al. [7]	Randomized Clinical Trials	41	QS-532	5–40 ns	5–6	1.5–2.5	1–3	12	24	VAS = 2.63 ± 1.06	46.0%
			QS-755	70 ns	3	6.0–8.0				VAS = 2.84 ± 1.11	
		29	PS-755	750 ps	2	5.56–6.37				VAS = 2.74 ± 1.05	
Shimbashi et al. [21]	Prospective	12	QS-694	25 ns	/	6	1–6	4–6	21	30% improve 51–75%, 16% improve 26–50%, 50% improve 0–25%	0
Shimbashi and Kojima [22]	Prospective	21	694	450 μs	/	17.5–27.5	2	12	20	24% good, 57% fair, 14% poor, 5% aggravated	64.7%
Gu et al. [9]	Retrospective	67	Fractional QS-694	/	7.1	5.0–5.5	≥ 1	3–4	35	100% improve ≥ 50%	0
		54	IPL 560	3–4 ms	15 × 35/ 15 × 8	13–15	≥ 1			88% improve ≥ 50%	
Wang et al. [24]	Prospective	48	QS-755	50–100 ns	3	7–17	1–10	16–24	39	31% improve > 75%, 23% improve 51–75%, 29% improve 26–50%, 17% improve $\le$ 25%	19.2%
Zhang et al. [25]	Prospective	471	QS-755	50–60 ns	3	5–17	1–9	12–144	/	29% improve ≥ 75%, 26% improve 50–74%, 23% improve 25–49%, 20% improve < 25%	/
Zhuang et al. [8]	Randomized Clinical Trials	21	QS-532	/	3	2.0–2.2	3	4	6	5% improve > 95%, 19% improve 75–95%, 33% improve 50–75%, 5% improve 25–50%, 5% improve < 25%	16.7%
		19	QS-1064		6	3.11–3.18	6	2		32% improve > 95%, 11% improve 75–95%, 26% improve 50–75%, 11% improve 25–50%	0
Kim et al. [19]	Prospective	4	QS-1064	/	7–7.5	2.4–2.5	12–24	2	24	25% nearly clean, 50% improve markedly, 25% improve moderately	0
Lin et al. [10]	Retrospective	52	QS-1064	/	5	3.6–4.0	1–5	8	54	10% complete, 23% excellent, 29% good, 25% fair, 13% poor	0
Baek et al. [15]	Prospective	35	QS-1064	/	7	2.2–2.4	20–50	1	12	69% improve > 95%, 26% improve 76–95%, 6% improve 51–75%	0
Kung et al. [20]	Prospective	2	PS-532	/	3–6	0.36–0.87	3–5	2–6	3	50% improve 75–94%, 50% improve 50–74%	0
Artzi et al. [11]	Retrospective Case Series	16	PS-532	/	4–5	0.8–1.6	1–4	4–8	9	31% improve > 95%, 25% improve 75–95%, 38% improve 50–75%, 6% improve < 25%	13.3%
Fitzpatrick et al. [16]	Prospective	16	510	300 ns	5	1–4	1–3	4	6	50% improve 100%, 13% improve 75%, 31% improve 50%, 6% improve 25%	0
Somyos et al. [23]	Prospective	16	511	20 ns	0.3	7–22	1–4	2	22	56% improve 90–100%, 38% improve 70–89%, 6% improve < 50%	0
Balaraman et al. [12]	Retrospective Case Series	4	1550	/	15	6–70 mJ	4–7	4–8	/	50% improved > 75%, 25% improved 50–75%, 25% improved < 25%	/
Kim et al. [18]	Prospective	6	QS-1064	/	2.6	1–1.2	3	4	6	66.7% improve > 95%, 16.7% improve 75–95%, 16.7% improve 50–75%	16.7%
		6	QS-532	/	7	2.6–3	6	2	6	16.7% improve > 95%, 16.7% improve 75–95%, 33.4% improve 50–75%, 33.4% improve 25–50%	33.4%
Alster [14]	Prospective	34	510	300 ns	5	2.0–4.0	6–8	4–14	12	Indistinguishable	0
Belkin et al. [13]	Retrospective Case Series	43	QS-1064/ QS-755/ QS-532	/	/	/	/	/	/	VAS = 2.86	/
		4	PS-755	/	/	/	/	/	/		

**Fig. 2:**
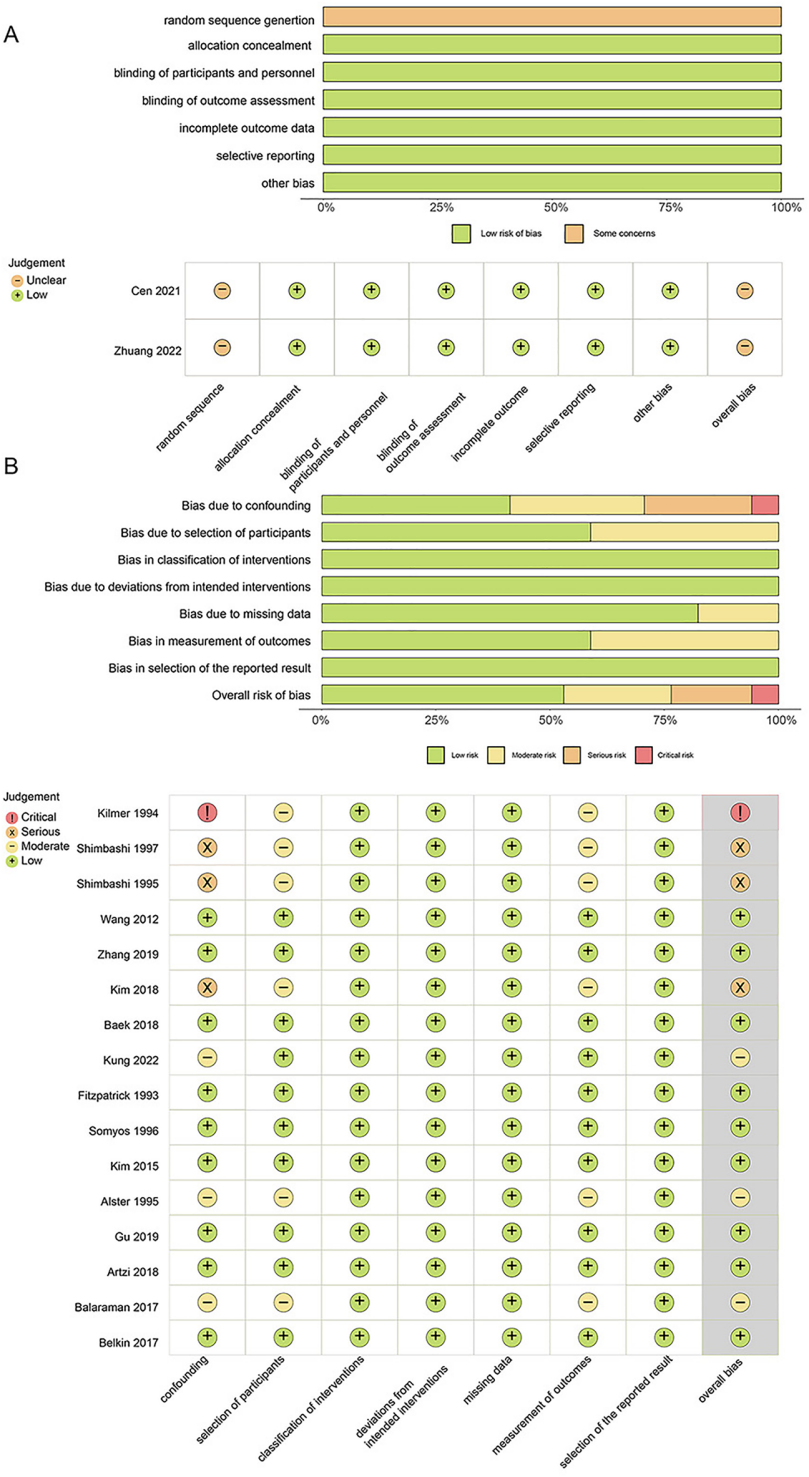
Results of the risk of bias assessment using RoB2 and ROBINS-I. **a** Bar chart overview and per-study risk of bias rating for RCT studies. **b** Bar chart overview and per-study risk of bias rating for observational studies

The risk of bias assessment results is presented in Fig. 2. According to the Cochrane risk of bias tool for randomized trials (RoB2), 2 RCTs were both rated as having some concerns only regarding the random sequence generation and were rated as having some concerns about the overall risk of bias [7]. For non-RCTs, the risk of bias in non-randomized studies of interventions (ROBINS-I) indicated that Kilmer et al. had a critical risk of bias due to the serious concerns regarding confounding factors and measurement of outcomes [17]. Shimbashi, T. et al. and Kim, H. et al. were rated as having serious risks of bias because of confounding factors [22].

### Efficacy

**Fig. 3:**
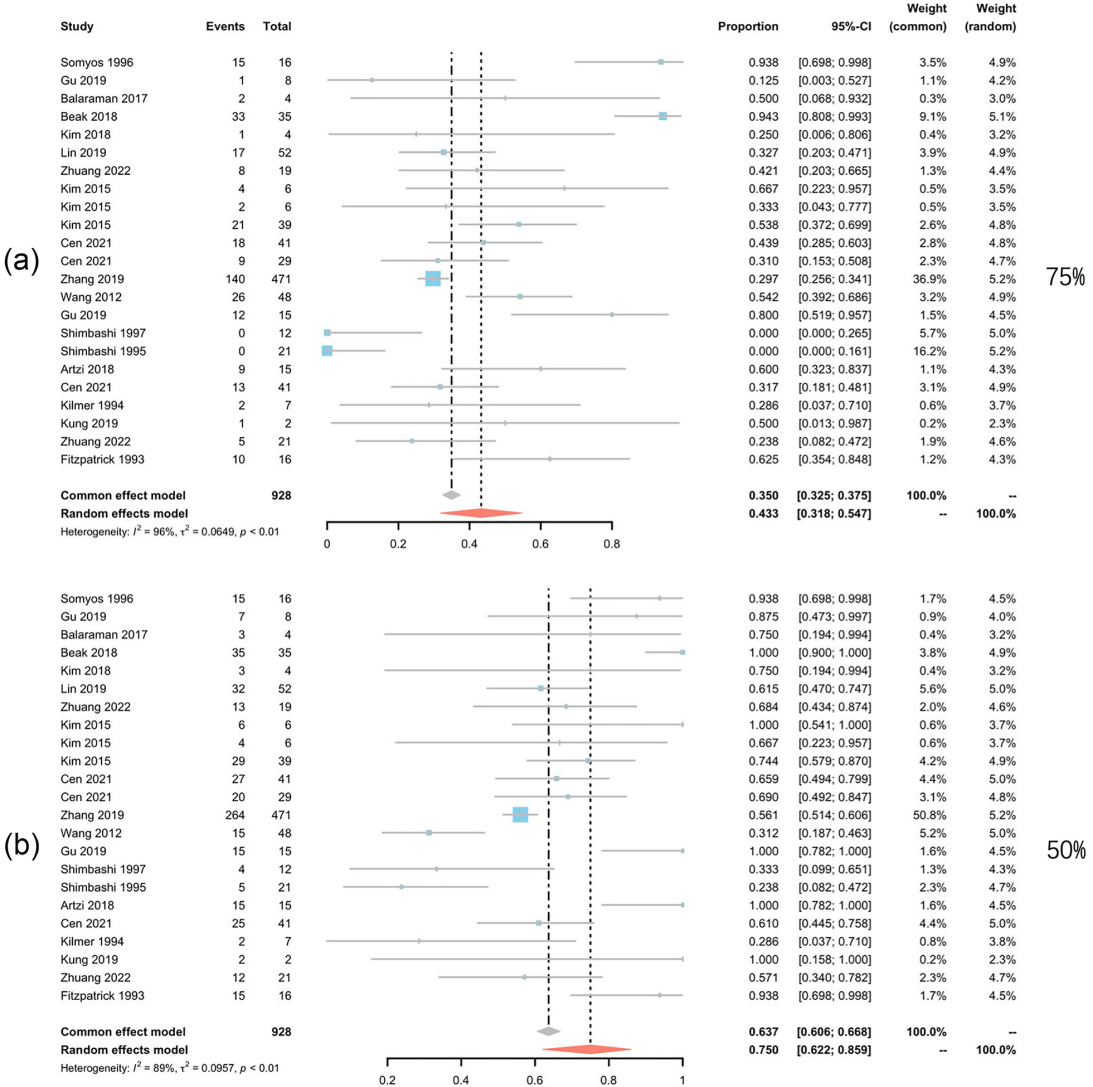
Forest plots for the clearance rate. **a** Clearance rate over 75%;**b** clearance rate over 50%

Data of 928 patients in 17 studies [15] were extracted to pool the clearance rate, and 349 patients from 10 studies were analyzed for recurrence rate [15]. According to the Grading of Recommendations, Assessment, Development and Evaluation (GRADE) system, all the results are rated as having high or moderate quality (Table 2). The percentage of patients whose CALMs clearance rate reached 75% was 43.3% (95% CI 31.8–54.7%, *I*^2^ = 96%), and the percentage of patients whose CALMs clearance rate reached 50% was 75% (95% CI 62.2–85.9%, *I*^2^ = 89%) (Fig. 3).

**Table 2:** GRADE table for this meta-analysis

Outcome	No. of studies (patients)	Risk of bias	Inconsistency	Indirectness	Imprecision	Publication bias	Quality of evidence
75% Clearance-overall	17 (928)	Not serious (23.5% had a high risk of bias)	Serious (high heterogeneity *I*^2^ = 96%)	Not serious	Not serious (95% CI 0.318–0.547)	Not serious (no evidence)	Moderate
75% Clearance-Q	11 (817)	Not serious (27.3% had a high risk of bias)	Serious (high heterogeneity *I*^2^ = 95%)	Not serious	Not serious (95% CI 0.299–0.559)	Not serious (no evidence)	Moderate
75% Clearance-NQ	8 (111)	Not serious (12.5% had a high risk of bias)	Serious (high heterogeneity *I*^2^ = 96%)	Not serious	Not serious (95% CI 0.209–0.677)	Not serious (no evidence)	Moderate
75% Clearance-1064	5 (155)	Not serious (20% had a high risk of bias)	Serious (high heterogeneity *I*^2^ = 90%)	Not serious	Not serious (95% CI 0.269–0.747)	Not serious (no evidence)	Moderate
75% Clearance-755	3 (589)	Not serious (0.0% had a high risk of bias)	Serious (high heterogeneity *I*^2^ = 77%)	Not serious	Not serious (95% CI 0.272–0.505)	Not serious (no evidence)	Moderate
75% Clearance-694	3 (48)	Serious (66.6% had a high risk of bias)	Serious (high heterogeneity *I*^2^ = 96%)	Not serious	Not serious (95% CI 0–0.775)	Not serious (no evidence)	Moderate
75% Clearance-532	6 (92)	Not serious (16.7% had a high risk of bias)	Not serious (low heterogeneity *I*^2^ = 38%)	Not serious	Not serious (95% CI 0.271–0.466)	Not serious (no evidence)	High
50% Clearance-overall	17 (928)	Not serious (23.5% had high risk of bias)	Serious (high heterogeneity *I*^2^ = 89%)	Not serious	Not serious (95% CI 0.622–0.859)	Not serious (no evidence)	Moderate
50% Clearance-Q	11 (817)	Not serious (27.3% had a high risk of bias)	Serious (high heterogeneity *I*^2^ = 96%)	Not serious	Not serious (95% CI 0.542–0.784)	Not serious (no evidence)	Moderate
50% Clearance-NQ	8 (111)	Not serious (12.5% had a high risk of bias)	Serious (high heterogeneity *I*^2^ = 87%)	Not serious	Not serious (95% CI 0.625–0.977)	Not serious (no evidence)	Moderate
50% Clearance-1064	5 (155)	Not serious (20% had a high risk of bias)	Serious (high heterogeneity *I*^2^ = 88%)	Not serious	Not serious (95% CI 0.583–0.937)	Not serious (no evidence)	Moderate
50% Clearance-755	3 (589)	Not serious (0% had a high risk of bias)	Serious (high heterogeneity *I*^2^ = 83%)	Not serious	Not serious (95% CI 0.389–0.714)	Not serious (no evidence)	Moderate
50% Clearance-694	3 (48)	Serious (66.6% had a high risk of bias)	Serious (high heterogeneity *I*^2^ = 97%)	Not serious	Not serious (95% CI 0.052–1.000)	Not serious (no evidence)	Moderate
50% Clearance-532	6 (92)	Not serious (16.7% had a high risk of bias)	Serious (high heterogeneity *I*^2^ = 87%)	Not serious	Not serious (95% CI 0.500–0.995)	Not serious (no evidence)	Moderate
Recurrence-overall	10 (349)	Not serious (30.0% had a high risk of bias)	Serious (high heterogeneity *I*^2^ = 88%)	Not serious	Not serious (95% CI 0.032–0.265)	Not serious (no evidence)	Moderate
Recurrence-1064	5 (155)	Not serious (20.0% had a high risk of bias)	Serious (high heterogeneity *I*^2^ = 72%)	Not serious	Not serious (95% CI 0–0.102)	Not serious (no evidence)	Moderate
Recurrence-755	2 (89)	Not serious (0.0% had a high risk of bias)	Serious (high heterogeneity *I*^2^ = 93%)	Not serious	Not serious (95% CI 0.011–0.660)	Not serious (no evidence)	Moderate
Recurrence-694	2 (33)	Serious (100.0% had a high risk of bias)	Not serious (low heterogeneity *I*^2^ = 0%)	Not serious	Not serious (95% CI 0.430–0.774)	Not serious (no evidence)	Moderate
Recurrence-532	3 (42)	Not serious (0.0% had a high risk of bias)	Not serious (low heterogeneity *I*^2^ = 0%)	Not serious	Not serious (95% CI 0.033–0.260)	Not serious (no evidence)	High
Hypopigmentation-overall	14 (951)	Not serious (14.3% had a high risk of bias)	Not serious (low heterogeneity *I*^2^ = 0%)	Not serious	Not serious (95% CI 0.004–0.020)	Not serious (no evidence)	High
Hypopigmentation-1064	5 (155)	Not serious (20.0% had a high risk of bias)	Not serious (low heterogeneity *I*^2^ = 26%)	Not serious	Not serious (95% CI 0.000–0.025)	Not serious (no evidence)	High
Hypopigmentation-755	4 (634)	Not serious (0.0% had a high risk of bias)	Not serious (low heterogeneity *I*^2^ = 16%)	Not serious	Not serious (95% CI 0.003–0.025)	Not serious (no evidence)	High
Hypopigmentation–532	3 (68)	Not serious (0.0% had a high risk of bias)	Not serious (low heterogeneity *I*^2^ = 25%)	Not serious	Not serious (95% CI 0.000–0.075)	Not serious (no evidence)	High
Hyperpigmentation-overall	14 (951)	Not serious (14.3% had a high risk of bias)	Not serious (low heterogeneity *I*^2^ = 0%)	Not serious	Not serious (95% CI 0.003–0.020)	Not serious (no evidence)	High
Hyperpigmentation-1064	5 (155)	Not serious (20.0% had a high risk of bias)	Not serious (low heterogeneity *I*^2^ = 0%)	Not serious	Not serious (95% CI 0.000–0.025)	Not serious (no evidence)	High
Hyperpigmentation-755	4 (634)	Not serious (0.0% had a high risk of bias)	Not serious (low heterogeneity *I*^2^ = 0%)	Not serious	Not serious (95% CI 0.002–0.022)	Not serious (no evidence)	High
Hyperpigmentation–532	3 (68)	Not serious (0.0% had a high risk of bias)	Not serious (low heterogeneity *I*^2^ = 32%)	Not serious	Not serious (95% CI 0.000–0.039)	Not serious (no evidence)	High

75% Clearance-Q, the ratio of 75% clearance rate by Q-switch laser treatment; 75% Clearance-NQ, the ratio of 75% clearance rate by non-Q-switch laser treatment; 75% Clearance-1064, the ratio of 75% clearance rate by 1064 nm laser treatment; 75% Clearance-755, the ratio of 75% clearance rate by 755 nm laser treatment; 75% Clearance-694, the ratio of 75% clearance rate by 694 nm laser treatment; 75% Clearance-532, the ratio of 75% clearance rate by 532 nm laser treatment; 50% Clearance-Q, the ratio of 50% clearance rate by Q-switch laser treatment; 50% Clearance-NQ, the ratio of 50% clearance rate by non-Q-switch laser treatment; 50% Clearance-1064, the ratio of 50% clearance rate by 1064 nm laser treatment; 50% Clearance-755, the ratio of 50% clearance rate by 755 nm laser treatment; 50% Clearance-694, the ratio of 50% clearance rate by 694 nm laser treatment; 50% Clearance-532, the ratio of 50% clearance rate by 532 nm laser treatment; Recurrence-1064, the recurrence rate of 1064 nm laser treatment; Recurrence-755, the recurrence rate of 755 nm laser treatment; Recurrence-694, the recurrence rate of 694 nm laser treatment; Recurrence-532, the recurrence rate of 532 nm laser treatment; Hypopigmentation-1064, the occurrence rate of hypopigmentation in 1064 nm treatment group; Hypopigmentation-755, the occurrence rate of hypopigmentation in 755 nm treatment group; Hypopigmentation-532, the occurrence rate of hypopigmentation in 532 nm treatment group; Hyperpigmentation-1064, the occurrence rate of hyperpigmentation in 1064 nm treatment group; Hyperpigmentation-755, the occurrence rate of hyperpigmentation in 755 nm treatment group; Hypoerpigmentation-532, the occurrence rate of hyperpigmentation in 532 nm treatment group

**Fig. 4:**
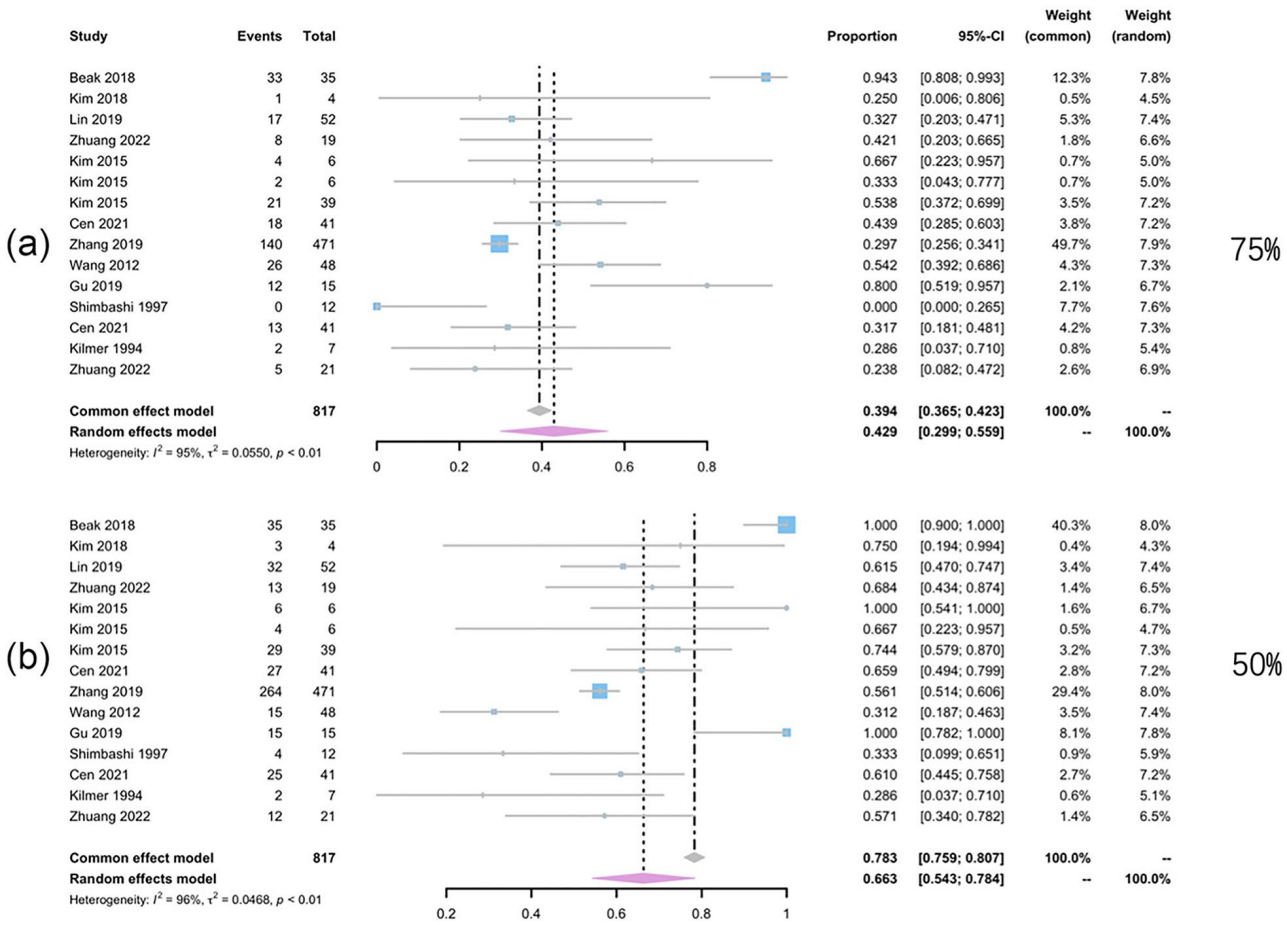
Forest plots for the clearance rate of Q-switch laser treatment. **a** Clearance rate over 75%; **b** clearance rate over 50%

**Fig. 5:**
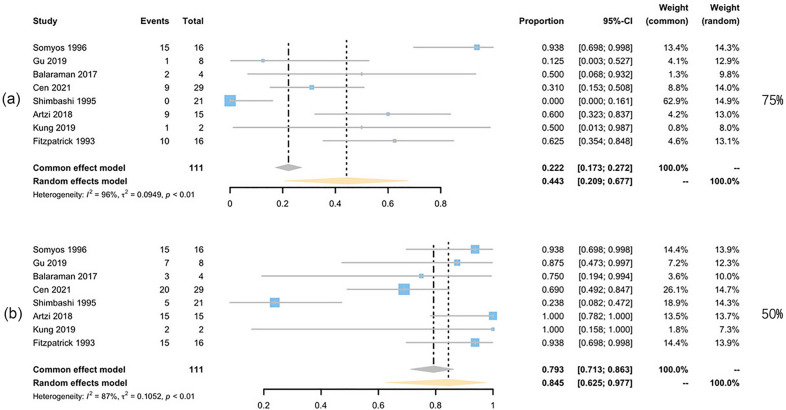
Forest plots for the clearance rate of Non-Q-switch laser treatment. **a** Clearance rate over 75%; **b** clearance rate over 50%

**Fig. 6:**
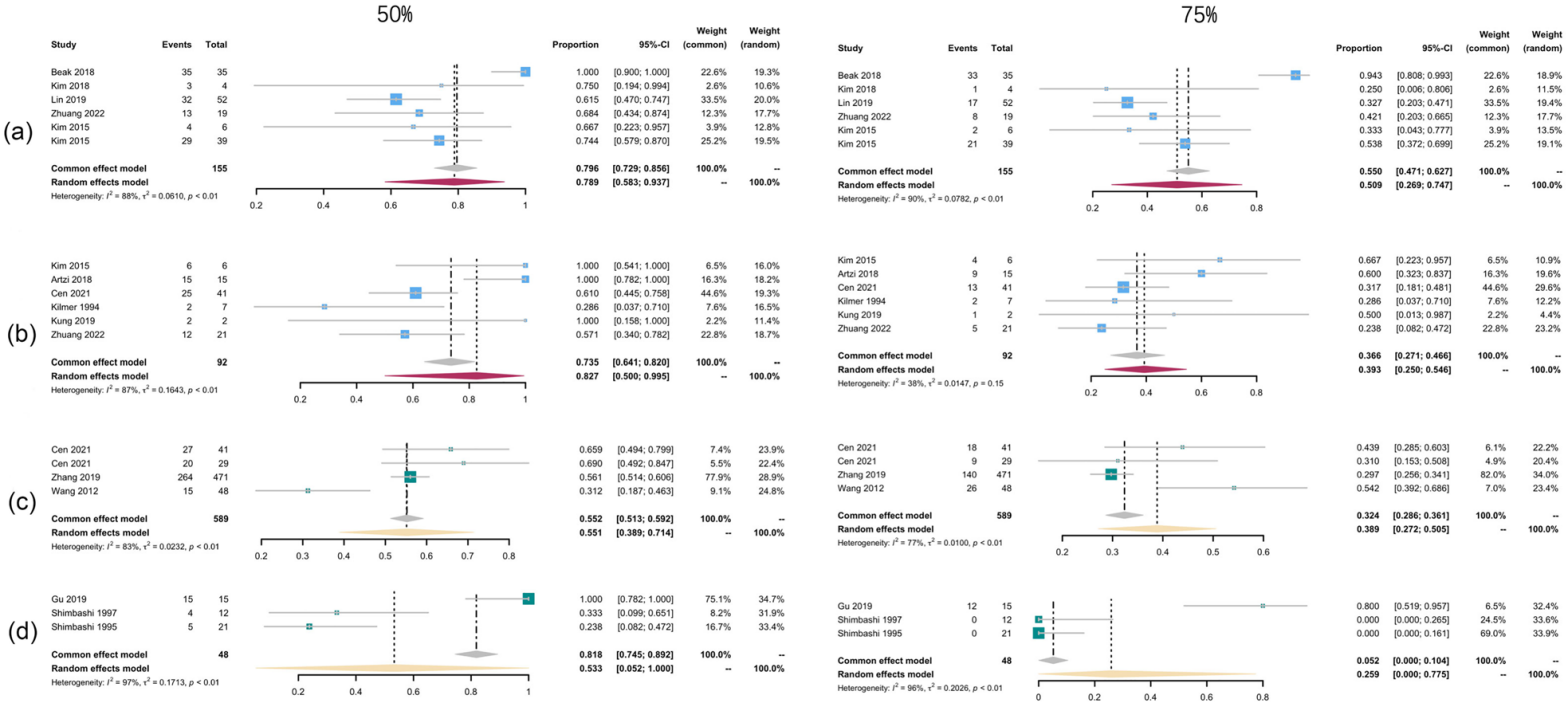
Forest plots for the clearance rate of 50% and 75% between wavelength groups. **a** 1064 nm laser treatment; **b** 532 nm laser treatment; **c** 755 nm laser treatment; **d** 694 nm laser treatment

**Fig. 7:**
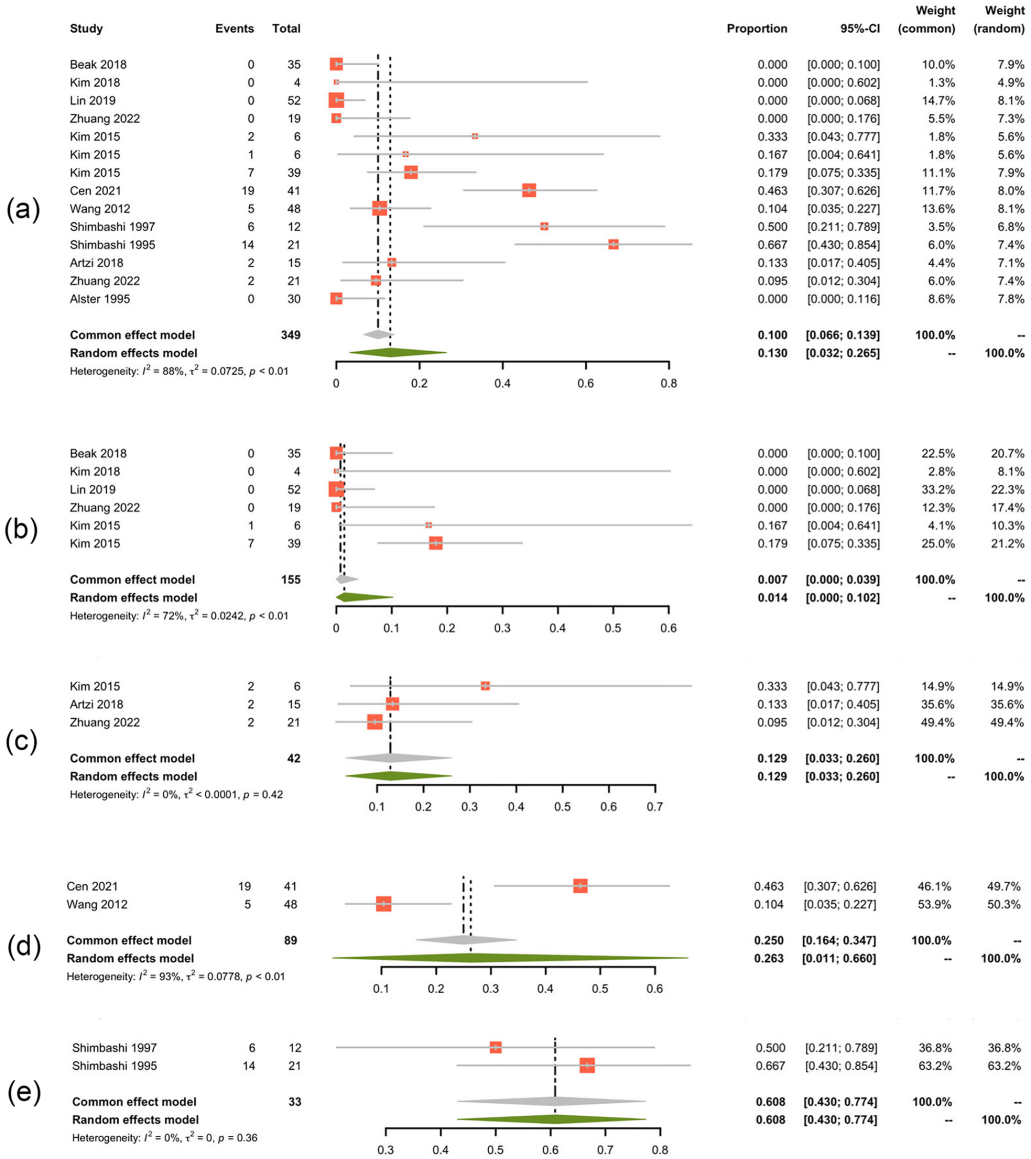
Forest plots for the recurrence rate. **a** Total recurrence rate; **b** 1064 nm laser treatment; **c** 532 nm laser treatment; **d** 755 nm laser treatment; **e** 694 nm laser treatment

Subgroup analysis was conducted between the Q-switched laser treatment (Fig. 4) and non Q-switched treatment group (Fig. 5). Two groups presented comparable efficacy when looking at the patients reaching 75% clearance (42.9% with *I*^2^ of 95% for Q-switched vs 44.3% with *I*^2^ of 96% for non Q-switched). But 84.5% of the patients receiving the non Q-switched laser presented clearance of 50% ~ 75%, which is higher than that of Q-switched group (66.3%). Subgroup analysis of different wavelengths lasers suggested that, 50.9% (95% CI 26.9–74.4%, *I*^2^ = 90%) of the patients treated with QS-1064-nm Nd:YAG laser achieved a 75% clearance rate, which was the highest among different wavelengths lasers treatment groups (Fig. 6). The 532 nm laser treatment turned out to be easier to take effect with 82.7% of the patients could present over 50% clearance (95% CI 50–99.5%), *I*^2^ = 87%). The overall pooled recurrence rate was 13% (95% CI 3.2–26.5%, *I*^2^ = 88%) (Fig. 7). Among different wavelength groups, the 1064 nm laser treatment presented the lowest recurrence rate 1.4%, and 694 nm laser treatment resulted in the highest recurrence rate of 60.8%.

### Side effects

**Fig. 8:**
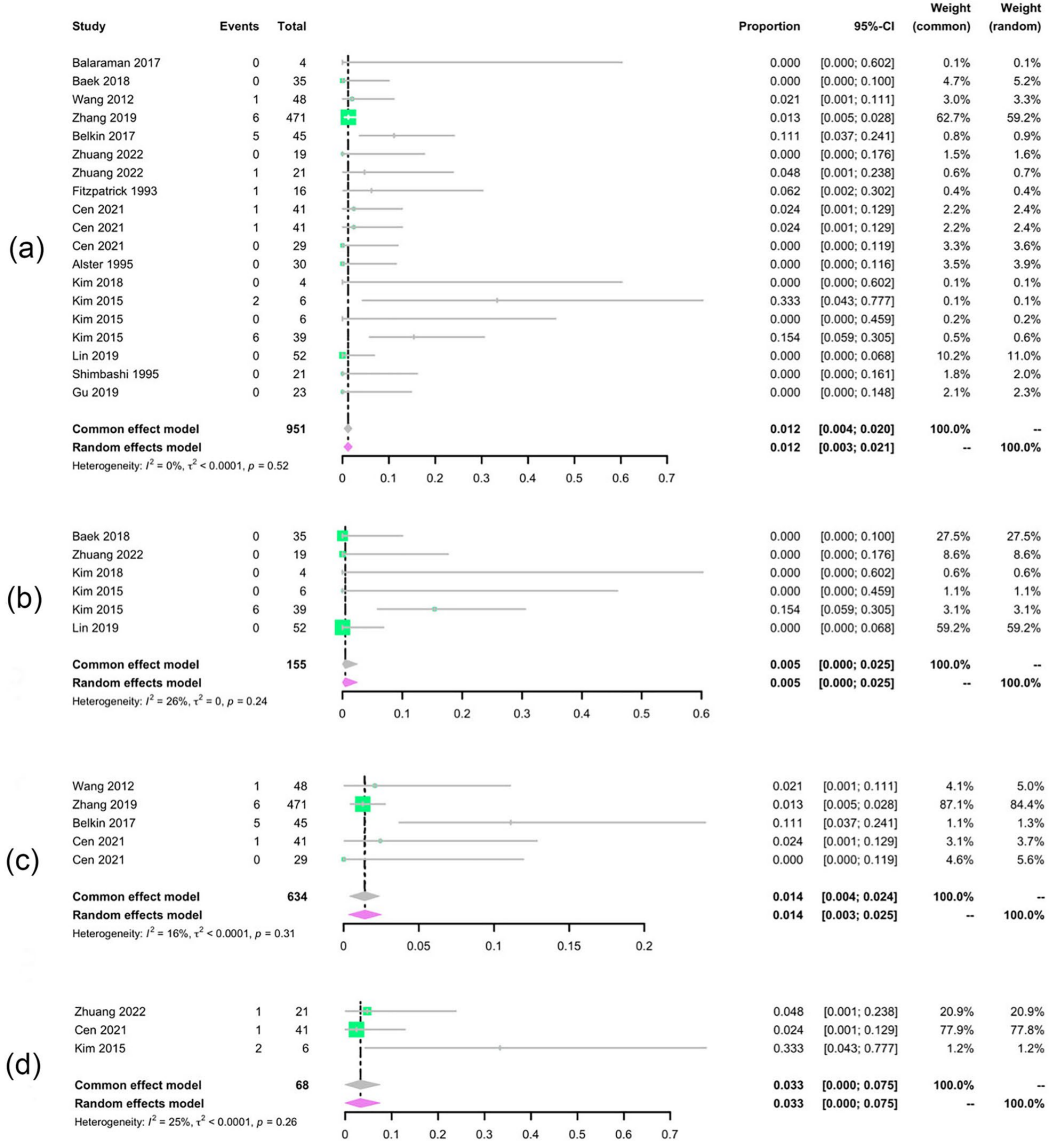
Forest plots for the hypopigmentation. **a** Total hypopigmentation; **b** 1064 nm laser treatment; **c** 755 nm laser treatment; **d** 532 nm laser treatment

**Fig. 9:**
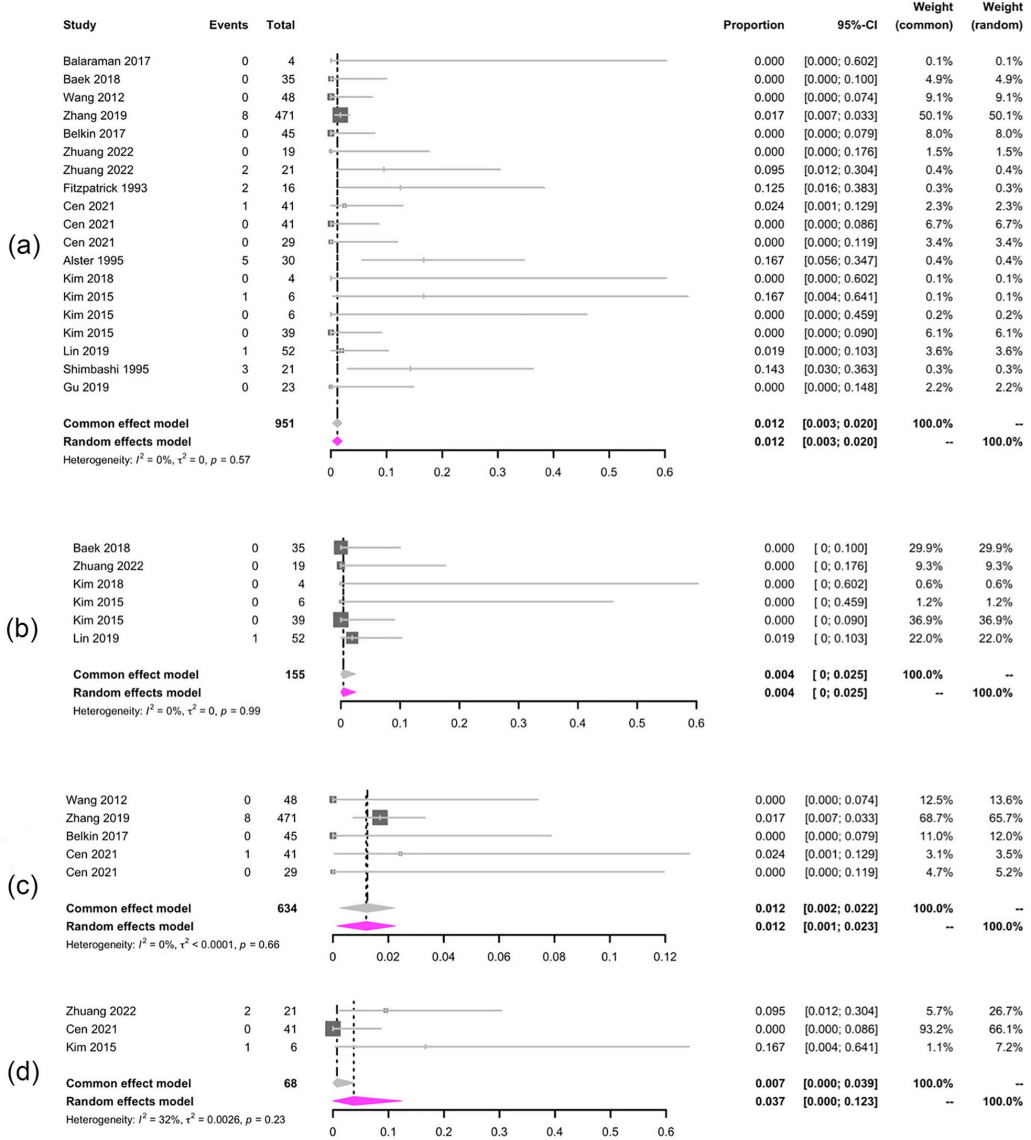
Forest plots for the hyperpigmentation. **a** Total hyperpigmentation; **b** 1064 nm laser treatment; **c** 755 nm laser treatment; **d** 532 nm laser treatment

The pooled hypopigmentation and hyperpigmentation rates were 1.2% (95% CI 0.3–2.1%, *I*^2^ = 0%) and 1.2% (95% CI 0.3–2%, *I*^2^ = 0%) respectively, which demonstrated an acceptable safety of laser treatment (Figs. 8, 9).

Among different wavelengths lasers, 1064 nm laser treatment presented the lowest hypopigmentation and hyperpigmentation rate of 0.5% (95% CI 0.0–2.5%, *I*^2^ = 26%) and 0.4% (95% CI 0.0–2.5%, *I*^2^ = 0%).

### Sensitivity analysis and publication bias

**Fig. 10:**
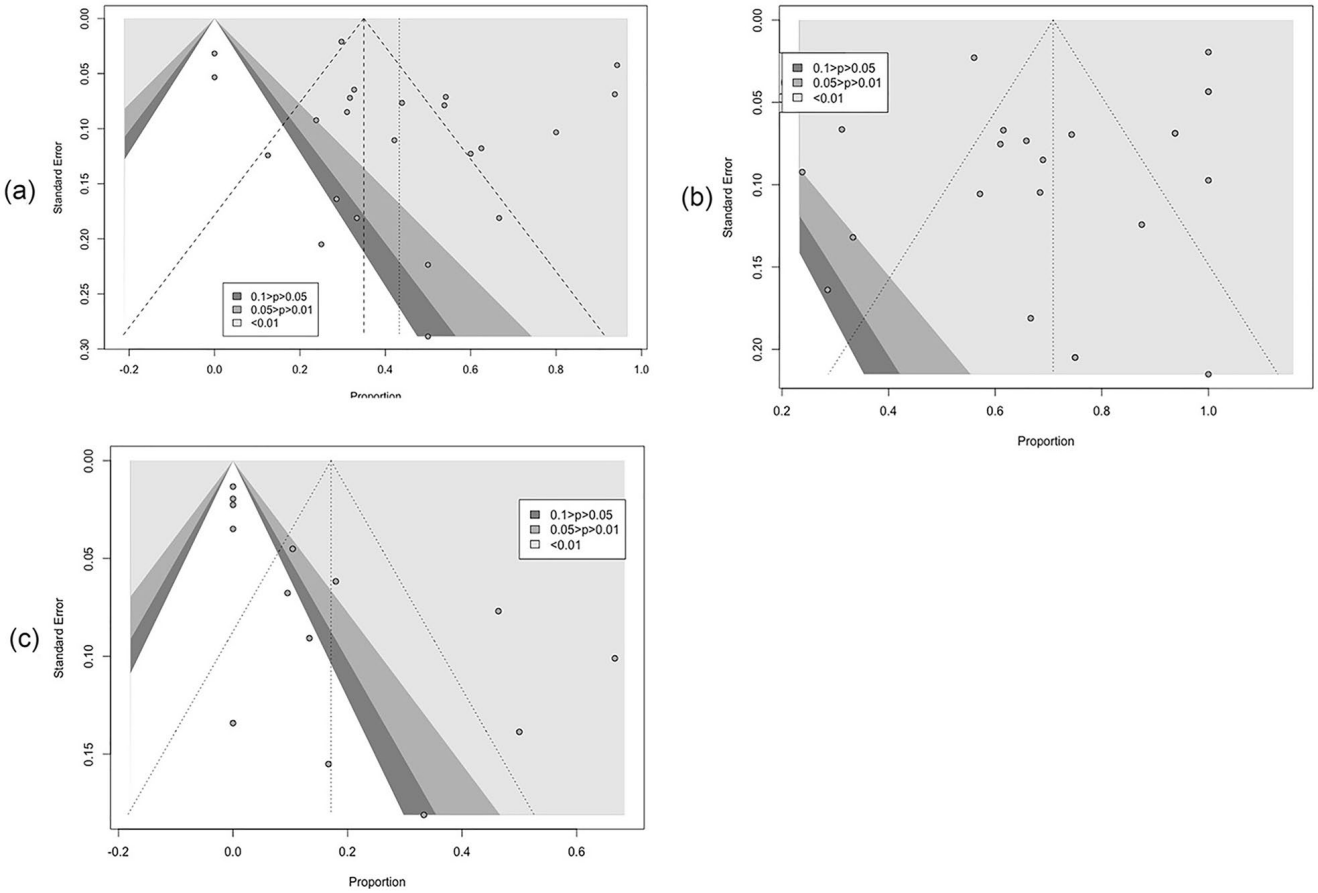
Funnel plots for publication bias based on **a** 75% clearance rate; **b** 50% clearance rate; **c** recurrence rate

**Fig. 11:**
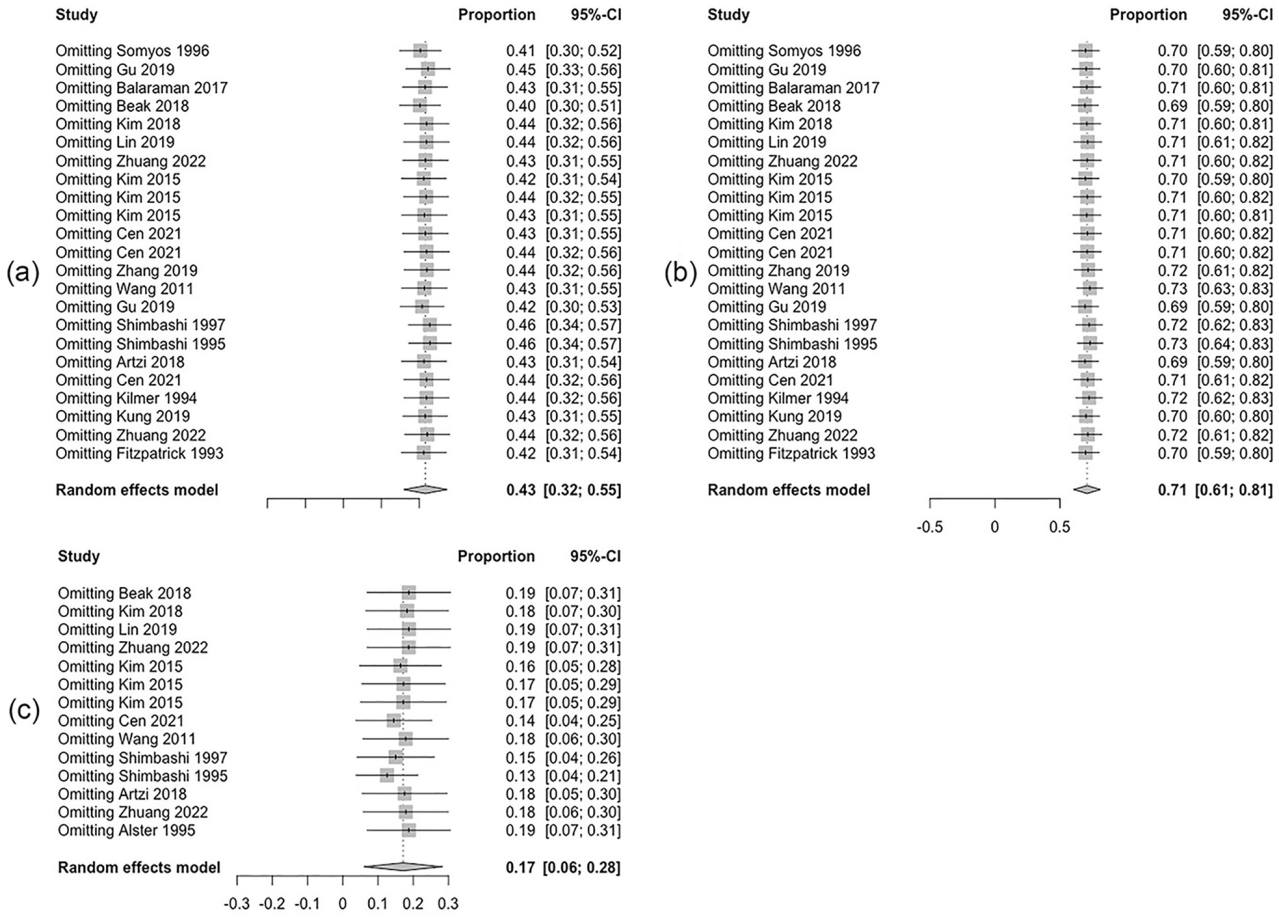
Sensitivity analysis based on **a** 75% clearance rate; **b** 50% clearance rate; **c** recurrence rate

Funnel plots were made based on a 75% clearance rate, 50% clearance rate, and the recurrence rate respectively, and are shown in Fig. 10. There was no evidence of publication bias. The result of the sensitivity analysis is presented in Fig. 11, none of the exclusion of individual studies changed the previous meta-analysis result, suggesting that the heterogeneity does not result from a single study.

## Discussion

The yielded result demonstrated the laser treatment without differentiation of wavelengths could reach a satisfactory result with 75% of patients with CALMs presenting a clearance rate of more than 50%. When looking at the efficancy between different wavelength subgroups, QS-1064-nm Nd:YAG laser exhibited the best treatment capability with minimal side effects. However, according to the experience of our center, intense pulsed light (IPL) treatment sometimes achieves better results than QS-1064-nm Nd:YAG laser treatment. We, therefore, believe that the results of this meta-analysis should be referred to with caution and taken into account in conjunction with clinical experience. Further clinical studies with larger sample sizes are warranted to resolve such disagreements. Interestingly, in Yuichi Yoshida study [26], the treatment of pigmented lesions with Neurofibromatosis Type I (NF1) by intense pulsed-radio frequency (IPL-RF) in combination with topical application of vitamin D3 ointment resulted in moderate to good improvement in 6 of 8 cases(75%). This study indicates laser irradiation in combination with the topical application of ointment would be useful as a new modality for refractory CALMs.

This meta-analysis had some limitations, and the results should be interpreted with caution. Firstly, the heterogeneity of pooling clearance data was high, even with the subgroup analysis conducted between different wavelength groups, which indicated that not only the wavelength of lasers affected treatment effects but also many other factors influence the therapeutic efficacy. One one hand, the efficacy of laser treatment is significantly dependent on the customization of the treatment protocol for each individual, which is based on the expert experience and judgment of the dermatologist rather than a standardized criterion. However, the diversity and heterogeneity of reported treatment parameters, including energy, spot size, treatment sessions, and interval time, have made it challenging to perform a thorough sub-group analysis. According to our clinical practice, low fluence, optimal spot size and optimal interval time laser treatment will achieve better treatment results and minimize side effects. Specifically, the low-fluence (LF) QS-1064-nm Nd:YAG laser treatment which had a controlled energy of 1.6–3.5 J/cm^2^, a large spot size of 6–8 mm with multiple-passed could reach the laser toning function [27] and avoided serious epidermal damage and adverse effects like hypopigmentation [30]. In Beak’s clinical trials [15], compared to other QS-1064 nm clinical trials, he selected a relatively medium spot size of 7 mm, lower fluence of 2.2–2.4 J/cm^2^, quite long treatment sessions of 20–50 weeks, and relatively shorter interval of 1 week. 69% of patients in his study improved by > 95%, 26% improved by 76–95%, and 6% improved by 51–75%, which were much better than other clinical trials’ results [18]. On the other hand, the intrinsic properties of CALMs, including their shape, size, location, color, and distribution, are believed to impact the effectiveness of treatment, thereby presenting a potential source of heterogeneity in results. While the shape of CALMs has been established as a crucial determinant of therapeutic outcomes, as evidenced by previous studies [18]. Specifically, CALMs with irregular margins (resembling the coast of Maine) have demonstrated better treatment response compared to those with clearly defined borders (resembling the coast of California) [8]. Nonetheless, the limited availability of initial data linking intrinsic features of CALMs with treatment outcomes precludes a subgroup analysis of their influence on treatment efficacy [18]. Several studies have suggested that smaller CALMs exhibit superior therapeutic responses compared to larger lesions [8]. However, the results have not achieved statistical significance in some investigations [15]. Similarly, while some studies have linked the brown color of CALMs to better treatment outcomes [8], others failed to establish any significant correlation [7]. Facial lesions have also been shown to exhibit better responses to treatment than those found in other areas of the body [18], although these results were also controversy [15]. It is also worth noting that the subjective visual evaluation of CALMs by different dermatologists may affect the assessment of therapeutic efficacy.

Secondly, the sample sizes of the included studies were too small to be divided into different age groups. Given that children had relatively thin skin and lightly colored lesions which may result in better treatment efficacy, further studies with patient-level data could enable more accurate analysis results. Thirdly, the pain was a relatively subjective side effect that was qualitatively but not quantitatively evaluated in resource data, so it was not included in the analysis.

In conclusion, the current systematic review and meta-analysis demonstrated the efficacy and side effects of laser treatment for patients with CLAMs. Among all wavelength lasers, QS-1064-nm Nd:YAG laser treatment resulted in the best clearance rate and the lowest recurrence, hypopigmentation, and hyperpigmentation rate while compared to other wavelength laser treatments. Further large-scale, randomized controlled trials are needed to confirm our current results.

## Materials and methods

The current meta-analysis was performed according to the recommendations of the latest “Preferred Reporting Items for Systematic Reviews and Meta-Analyses” (PRISMA 2020) [32]. And it was registered in the “International Prospective Register of Systematic Reviews” (PROSPERO) in 2022 (ID: CRD42022339049) and the detailed prespecified protocol is available upon request.

### Systematic literature search

We conducted a systematic literature search of three primary databases, including MEDLINE, Embase, and Web of Science to retrieve the articles published from the respective database initiation until April 11 2023. The exemplary search strategies were presented in supplement 1. Articles were included only if they were human studies published in English with full-text descriptions. Additionally, reference lists from retrieved articles were examined to identify relevant studies. Two independent reviewers determined the final inclusion of articles; when this failed, any disagreement was resolved by discussion.

### Inclusion criteria and exclusion criteria

Two reviewers screened and identified the search findings for potentially eligible studies. The inclusion criteria were as follows: (1) patients diagnosed with CALMs; (2) clear documentation of the intervention; (3) original articles reporting data on the clinical response or occurrence of adverse events in laser treatment; (4) studies reported in the English language; (5) When multiple studies were published by the same institution or authors, either the higher-quality study or the most recent publication was included.

Following studies were excluded:(1) abstracts, letters, expert opinions, and reviews; (2) studies with no reported outcomes of interest; (3) studies with insufficient data to extract; (4) data on pigmented lesions other than CALMs; (5) studies that report treatment response using the mean score of the degree of subject improvement rather than the specific number of subjects who achieved different degrees of improvement.

Two independent reviewers determined the final inclusion of articles; a third author adjudicated when this failed.

### Outcomes measured

The clinical response and recurrence and the occurrence of any types of adverse events were pooled and measured to evaluate the efficacy and safety of laser therapy. To measure the clinical response, the number of subjects reaching excellent (75% ~ clearance) and good (50–75% clearance) improvement were extracted from original studies. Adverse events of our interests include hypopigmentation and hyperpigmentation. The data on adverse events were extracted from subjects of any reported pigmented epidermal lesions undergone laser therapy rather than restricted to CALMs.

### Data extraction and quality assessment

One independent reviewer (D. W.) extracted the data using standardized forms and another reviewer checked the collected data. Any disagreements were resolved by discussion. The recorded data from the selected study included: (1) study characteristics (author, year of publication, institution, study design); (2) patient characteristics (patient number, age, gender, inclusion criteria, Fitzpatrick skin type, type of pigmented epidermal lesions, lesion location); (3) treatment protocol (type of lasers, treatment sessions, intervals, fluence); (4) clinical response; (5) type and occurrence of adverse events (AEs).

We assessed the risk of bias in RCTs using the revised RoB2 [33]. For non-randomized observational studies, the ROBINS-I tool was used to assess the risk of bias [34]. Two reviewers conducted the assessments independently (D.W. and ZZ.G.). Disagreements were resolved by recruiting a third author to attain consensus.

The overall quality of evidence for each outcome across the included studies was assessed using the GRADE system [35]. In this system, the quality of evidence was initially evaluated as “high”. After which, the quality may downgrade to moderate, low, or very low based on the criteria including risks of bias, inconsistencies, indirectness, imprecision, and publication bias [36].

### Statistical analysis

R version 4.2.0 and the R package ‘meta’ were used for performing the meta-analysis and generating the forest plots (R Foundation for Statistical Computing) [37]. Rates of patients reaching two cut-off treatment results, namely, 50% clearance and 75% clearance with a corresponding 95% confidence interval (CI) were calculated to perform the treatment efficacy. The pooled effects were calculated using both common-or random-effects models. Heterogeneity was evaluated by I^2^ with p < 0.1 taken as significant [38]. If the test yielded an *I*^2^ value > 50%, the random-effects analysis should be adopted. Sensitivity analyses were also performed by excluding individual studies from the data set to analyze their relative effects on the overall pooled estimates. Funnel plots were constructed to evaluate the potential publication bias.

## Data Availability

Not applicable.

## References

[1] Hirbe AC, Gutmann DH (2014). Neurofibromatosis type 1: a multidisciplinary approach to care. Lancet Neurol.

[2] Roberts AE, Allanson JE, Tartaglia M, Gelb BD (2013). Noonan syndrome. Lancet.

[3] Anderson S (2020). Café au Lait Macules and Associated Genetic Syndromes. J Pediatr Health Care.

[4] Grossman MC, Anderson RR, Farinelli W, Flotte TJ, Grevelink JM (1995). Treatment of cafe au lait macules with lasers. A clinicopathologic correlation. Arch Dermatol.

[5] Rauen KA (2013). The RASopathies. Annu Rev Genomics Hum Genet.

[6] Anderson RR, Parrish JA (1983). Selective photothermolysis: precise microsurgery by selective absorption of pulsed radiation. Science.

[7] Cen Q, Gu Y, Luo L, Shang Y, Rao Y, Zhu J (2021). Comparative effectiveness of 755-nm picosecond laser, 755- and 532-nm nanosecond lasers for treatment of Café-au-Lait Macules (CALMs): a randomized, split-lesion clinical trial. Lasers Surg Med.

[8] Zhuang Y, Huang M, Shen J, Wang L, Yang L, Jiang A (2022). Comparison of the efficacy and safety between a low-fluence 1064-nm Q-switched neodymium-doped yttrium aluminum garnet laser and a conventional Q-switched 532-nm laser for the treatment of cafe-au-lait macules in 40 Chinese children: a prospective, randomized, parallel-controlled, evaluator-blinded trial. Lasers Med Sci.

[9] Gu T, Yuan J, Zhang Y, Li YH, Wu Y, Gao XH (2021). A retrospective study of FQSRL and IPL in the treatment of Café-au-lait macule. J Dermatol Treat.

[10] Lin Y, Liu HX, Shi WH, Wang HX, Geng JH, Guo X (2019). Preliminary experience of the Q-switched 1064-nm neodymium:yttrium aluminum garnet laser in the treatment of Café-au-lait macules. J Eur Acad Dermatol Venereol.

[11] Artzi O, Mehrabi JN, Koren A, Niv R, Lapidoth M, Levi A (2018). Picosecond 532-nm neodymium-doped yttrium aluminium garnet laser-a novel and promising modality for the treatment of café-au-lait macules. Lasers Med Sci.

[12] Balaraman B, Ravanfar-Jordan P, Friedman PM (2017). Novel use of non-ablative fractional photothermolysis for café-au-lait macules in darker skin types. Lasers Surg Med.

[13] Belkin DA, Neckman JP, Jeon H, Friedman P, Geronemus RG (2017). Response to laser treatment of café au lait macules based on morphologic features. JAMA Dermatol.

[14] Alster TS (1995). Complete elimination of large café-au-lait birthmarks by the 510-nm pulsed dye laser. Plast Reconstr Surg.

[15] Baek JO, Park IJ, Lee KR, Ryu HR, Kim J, Lee S (2018). High-fluence 1064-nm Q-Switched Nd:YAG laser: Safe and effective treatment of café-au-lait macules in Asian patients. J Cosmet Dermatol.

[16] Fitzpatrick RE, Goldman MP, Ruiz-Esparza J (1993). Laser treatment of benign pigmented epidermal lesions using a 300 nsecond pulse and 510 nm wavelength. J Dermatol Surg Oncol.

[17] Kilmer SL, Wheeland RG, Goldberg DJ, Anderson RR (1994). Treatment of epidermal pigmented lesions with the frequency-doubled Q-switched Nd:YAG laser. A controlled, single-impact, dose-response, multicenter trial. Arch Dermatol.

[18] Kim H-R, Ha J-M, Park M-S, Lee Y, Seo Y-J, Kim C-D (2015). A low-fluence 1064-nm Q-switched neodymium-doped yttrium aluminium garnet laser for the treatment of cafe-au-lait macules. J Am Acad Dermatol.

[19] Kim J, Hur H, Kim YR, Cho SB (2018). Treatment of café-au-lait macules with a high-fluenced 1064-nm Q-switched neodymium:yttrium aluminum garnet laser. J Cosmet Laser Ther.

[20] Kung KY, Shek SY, Yeung CK, Chan HH (2019). Evaluation of the safety and efficacy of the dual wavelength picosecond laser for the treatment of benign pigmented lesions in Asians. Lasers Surg Med.

[21] Shimbashi T, Kamide R, Hashimoto T (1997). Long-term follow-up in treatment of solar lentigo and café-au-lait macules with Q-switched ruby laser. Aesthetic Plast Surg.

[22] Shimbashi T, Kojima T (1995). Ruby laser treatment of pigmented skin lesions. Aesthetic Plast Surg.

[23] Somyos K, Boonchu K, Somsak K, Panadda L, Leopairut J (1996). Copper vapour laser treatment of café-au-lait macules. Br J Dermatol.

[24] Wang Y, Qian H, Lu Z (2012). Treatment of café au lait macules in Chinese patients with a Q-switched 755-nm alexandrite laser. J Dermatol Treat.

[25] Zhang B, Chu Y, Xu Z, Sun Y, Li L, Han X (2019). Treatment of Café-Au-Lait spots using Q-switched Alexandrite laser: analysis of clinical characteristics of 471 children in Mainland China. Lasers Surg Med.

[26] Yoshida Y, Sato N, Furumura M, Nakayama J (2007). Treatment of pigmented lesions of neurofibromatosis 1 with intense pulsed-radio frequency in combination with topical application of vitamin D3 ointment. J Dermatol.

[27] Chan NP, Ho SG, Shek SY, Yeung CK, Chan HH (2010). A case series of facial depigmentation associated with low fluence Q-switched 1,064 nm Nd:YAG laser for skin rejuvenation and melasma. Lasers Surg Med.

[28] Herd RM, Dover JS, Arndt KA (1997). Basic laser principles. Dermatol Clin.

[29] Wattanakrai P, Mornchan R, Eimpunth S (2010). Low-fluence Q-switched neodymium-doped yttrium aluminum garnet (1,064 nm) laser for the treatment of facial melasma in Asians. Dermatol Surg.

[30] Polnikorn N (2008). Treatment of refractory dermal melasma with the MedLite C6 Q-switched Nd:YAG laser: two case reports. J Cosmet Laser Ther.

[31] Ara G, Anderson RR, Mandel KG, Ottesen M, Oseroff AR (1990). Irradiation of pigmented melanoma cells with high intensity pulsed radiation generates acoustic waves and kills cells. Lasers Surg Med.

[32] Page MJ, Moher D, Bossuyt PM, Boutron I, Hoffmann TC, Mulrow CD (2021). PRISMA 2020 explanation and elaboration: updated guidance and exemplars for reporting systematic reviews. BMJ.

[33] Sterne JAC, Savović J, Page MJ, Elbers RG, Blencowe NS, Boutron I (2019). RoB 2: a revised tool for assessing risk of bias in randomised trials. BMJ.

[34] Sterne JA, Hernán MA, Reeves BC, Savović J, Berkman ND, Viswanathan M (2016). ROBINS-I: a tool for assessing risk of bias in non-randomised studies of interventions. BMJ.

[35] Guyatt G, Oxman AD, Akl EA, Kunz R, Vist G, Brozek J (2011). GRADE guidelines: 1. Introduction-GRADE evidence profiles and summary of findings tables. J Clin Epidemiol.

[36] Balshem H, Helfand M, Schünemann HJ, Oxman AD, Kunz R, Brozek J (2011). GRADE guidelines: 3. Rating the quality of evidence. J Clin Epidemiol.

[37] RCoreTeam (2022). R: A language and environment for statistical computing.

[38] Higgins JP, Thompson SG, Deeks JJ, Altman DG (2003). Measuring inconsistency in meta-analyses. BMJ.

